# Accomplishing Intergroup Relations in Group Homes: A Discursive Analysis of Professionals Talking About External and Internal Stakeholders

**DOI:** 10.3389/fpsyg.2022.784345

**Published:** 2022-03-22

**Authors:** Marzia Saglietti, Filomena Marino

**Affiliations:** ^1^Department of Education, University of Bologna, Bologna, Italy; ^2^Department of Social and Developmental Psychology, Sapienza University of Rome, Rome, Italy

**Keywords:** intergroup relations, group homes, ethnographic interview, communities of practices, discourse analysis, ingroup bias

## Abstract

Focusing on one of the most studied dimensions of Social Psychology, i.e., intergroup relations, this study analyzes its discursive accomplishment in a specific group-based intervention, i.e., the talk and work of an Italian group home, i.e., a small alternative care facility hosting a group of out-of-home children. Particularly, we focused on the fictionally called “Nuns’ Home,” a group home previously investigated for its ethnocentric bias, and its intergroup relations with “inside” and “outside” groups, such as schools, biological families, and social services. By combining a qualitative and quantitative approach in analyzing one audio-recorded ethnographic interview with the whole team of professionals, we aimed at accounting for the multitude of internal and external stakeholders that participants refer to, analyzing the discursive accomplishment of ingroup and outgroup in talk-in-interaction and investigating ingroup bias and group qualification. To do so, we detected social categorization markers and qualifying devices that participants rely on when referring to groups. Results show that, among the numerous groups recognized, participants co-construct intergroup relations and ingroup bias implying negative assessment over external groups. Being different from traditional laboratory studies illustrating substantial contraposition between ingroup and outgroup, our qualitative analysis reveals the multitude of groups by which the ingroup is formed and their internal fragmentation. To conclude, we discussed the implications of qualitatively studying intergroup relations in group homes and indicated future lines of research.

## Introduction

One of the most prominent theories in Social Psychology is the social identity theory (SIT, Tajfel and Turner, 1985), a framework for investigating group processes and intergroup relations (Haslam et al., 2010). It is defined as “that part of an individual’s self-concept which derives from his knowledge of his membership of a social group (or groups) together with the emotional significance attached to that membership” (Tajfel, 1974), SIT has been studied with reference to the *ingroup bias* (Turner, 1975; Hogg and Terry, 2000), i.e., the preference for one’s own group (ingroup) in opposition to a group that one does not belong to (outgroup), and the social categorization theory (SCT, Turner et al., 1987), i.e., the cognitive representation of oneself with reference to group belonging. This theoretical framework has been studied by a series of experiments known as “minimal group studies,” in which laboratory participants casually assigned to experimental groups were asked to make the decision based on group belonging. Experimental results show that we positively judged the ingroup by referring to positive internal causes and prototypes – producing normative behaviors, cohesion, cooperation, and altruism (Hogg and Terry, 2000), and conversely, we negatively judged the outgroup, due to our cognitive ethnocentric bias, known as “fundamental attribution error” (Heider, 1958; Ross, 1977), partially explaining the development of prejudices and stereotypes.

However, this laboratory body of research privileged the study of social categories and large groups involving individuals who were not usually in interaction, failing to address social investigations with “natural” groups, i.e., non-artificial and historically based individuals connected by ordinary relationships (Zucchermaglio and Talamo, 2000). Bridging this gap, the qualitative line of group research (Edwards, 1995; Engeström and Middleton, 1996; Alby and Zucchermaglio, 2008) contributes by focusing on social and discursive interaction of “real-life” communities of practices (Lave and Wenger, 1991 p. 98): “a set of relations among persons, activity, and world, over time.” Therefore, intergroup relationships appear to be more complex than the sole ingroup-outgroup opposition, defining social identity as a “complex rhetorical-argumentative manipulation of one own’s (and others’) social positioning in a group, depending on variables such as the participants’ role and the topic of discursive interaction” (Zucchermaglio and Talamo, 2000, p. 524; on this topic, also refer to Wilson and Zeitlyn, 1995; Zucchermaglio and Talamo, 2000; Zucchermaglio, 2005; Alby and Zucchermaglio, 2008; Zucchermaglio and Alby, 2011; Brito Rivera et al., 2021; Fantasia et al., 2021. Membership categorization studies of Conversational Analysis (Sacks, 1972, 1992; Stokoe, 2012) contribute to the implementation of this body of research, showing that during the conversation, we frequently ascribed people to certain identity categories “by reference of either an explicit mentioning of the category term (*labeling*), or a *description* of actions or ‘predicates’ *bounded* to that category” (Fantasia et al., 2021, p. 452; also refer to Fasulo and Zucchermaglio, 2002; Benwell and Stokoe, 2011; Stokoe, 2012).

Particularly, interesting for this body of “*in vivo*” investigation of natural communities of practices can be group homes, i.e., alternative care settings temporarily recovering children who have experienced severe hardships (Rutter, 2000; Sellers et al., 2020). As group-based care intervention (Lee and Barth, 2011), group homes *host groups* of children, are, in fact, *organized in groups*, *managed by groups* (either residents or coordinated in shifts), and they deliver their intervention in *forms of a group care milieu* (Whittaker et al., 2016). Unfortunately, this intensive group-based work has been very little investigated so far, and even less (theoretical and empirical) emphasis has been given to their intergroup relations with “outside” groups, such as schools, biological families, social services, representing crucial interlocutors for children’s wellbeing. Bridging this gap and drawing on emergent literature focused on the discursive accomplishment of intergroup relations, we drew on a single-case study of an Italian group home. Our aims were as follows:

• accounting the multitude of internal and external stakeholders that participants refer to;
• analyzing the discursive accomplishment of ingroup and outgroup;
• investigating ingroup bias and group qualification.

Belonging to a broader dataset focusing on many discursive maneuvers this group relies on (Saglietti and Marino, forthcoming), this study corroborates the qualitative literature on group research by enlarging the empirical and analytical body of research on the discursive accomplishment of intergroup relations of “natural” communities of practices.

## Materials and Methods

### The Research Project

This study is part of a larger Ph.D. ethnographic project aimed at analyzing the everyday talk and work of three Italian group homes based in Rome (Saglietti, 2012; Saglietti and Zucchermaglio, 2021). The researcher’s access was negotiated with the local Social Services manager as well as with the professionals working in the selected settings (i.e., managers, social educators, psychologists, supervisors, and volunteers) and children hosted at the time of research. This study received approval from the University Ethics Committee, from the local Social Services of the Municipality of Rome (IT), and from the deputy public prosecutor of the local Juvenile Court. Professionals and tutors of children signed a written informed consent prior to data collection, according to the Italian Law concerning data protection and privacy. For the purpose of this study, we focused on a single facility, fictionally called “Nuns’ Home.” In previous comparative analyses based on organizational and conversational data (Saglietti, 2010, 2012; Saglietti and Zucchermaglio, 2021), it has proven to be based on ethnocentric biases and closeness to external stakeholders.

### Context and Participants

“Nuns’ Home” belongs to a Catholic female congregation. Previously hosting an orphanage, it is located in a building that, after the 149/2001 Italian Law, has been divided into two separate units. “Nuns’ Home” is one of them. At the moment of the research, this group home-hosted 7 children (4 boys and 3 girls, from 4 to 13 years old). Thereby, the professionals involved were three resident nuns (within them, the general manager), one non-resident educator (working on weekday afternoons), and one psychologist (offering in-house child psychological support).

### Data Collection, Selection, and Analysis

The research gathering conducted by the first author of this study took place from May 2007 to May 2009. The author collected ethnographic pen-to-paper participant observations of daily activities, shadowing of social educators and managers, audio-recordings of the staff’s weekly meetings, video-recorded dinnertime interactions, and two audio-recorded ethnographic interviews: one with resident nuns, and one with all team members. For this study, we focused on the latter. As a means to give voice to participants without fixing an *a priori* ideal interview structure and numbers of encounters (Serranò and Fasulo, 2011), the ethnographic interview built up discussion around specific issues that emerged during ethnographic observations, and particularly: (1) “Nuns’ Home” historical paths and challenges; (2) aims of the group home; (3) organizational management and everyday interaction; (4) relationships with external groups; and (5) views of professionals on strengths and limits about their interventions.

The ethnographic interview lasted 1 h and 51 min, and it was fully transcribed *verbatim*. In the first step of our analytical procedure, we listened multiple times to the original audio recordings and reread the transcripts, adjusting and finalizing the transcription process. In this phase, we also underlined the references that professionals made to intergroup relations, keeping in mind that “they are not preexisting outside discourse, but are ‘talked into being,’ that is, produced by the members to the interaction and can therefore be analyzed” (Maheux-Pelletier and Golato, 2008, p. 690). To investigate intergroup relation discursive accomplishment, for the purpose of this study, we focused exclusively on two discursive devices that we detected during this analytical phase, namely, social categorization markers and qualifying devices.

In line with previous research, we identified “social categorization markers” as any *group-referring expression* (GRE), i.e., collective nominal that refers to “a set of people, whether it be the addressee[s], (…) other participant[s], or absent part[ies]” (Wilson and Zeitlyn, 1995, p. 70) and pronominal markers (Zucchermaglio and Talamo, 2000; Zucchermaglio, 2005) that “are likely to be especially powerful influences in social cognition and perception. When these terms are used in reference to people, they are linked to one of the most basic decisions in person perception: the cognitive categorization of people into one’s ingroup or outgroup” (Perdue et al., 1990, p. 475). To investigate group qualification, with the term “qualifying devices,” we intended any adjective that describes the qualities of a group of people, usually located next to nouns, pronouns, or locutions referring to groups, implying assessment.

Taking into account the many implications and limits of coding a conversation (Schegloff, 2010; Steensig and Heinemann, 2015; Stivers, 2015) and considering the consequential nature and ambiguous delimitation of any coding definition, we nevertheless agreed on a codify and counting strategy. It has, therefore, been based on the following operations: (1) items were counted as phenomena (and not by means of their turn occupation); (2) each item was counted independently from its sequential location (i.e., we counted the exact number of items, even if they co-occurred in the same turn); (3) internal stakeholders were assigned to the ‘‘ingroup’’ category, i.e., groups that are physically operating within ‘‘Nuns’ Home’’, in the present, past, or (potential) future, and external stakeholders were assigned to the ‘‘outgroup,’’ i.e., groups that operate outside ‘‘Nuns’ Home’’; (4) each item was assigned to either ingroup or outgroup, based on the reference that participants used during the interview. We excluded from our dataset professionals’ implicit references (i.e., implicit pronominal references^1^) and singular expressions. Independently from their pragmatic function, we considered -- and consequently counted -- GREs, with reference to (a) *pronouns* used for referring to groups^2^ and (b) *common nouns*, i.e., generic names for groups of people. Within this last category and with reference to the family-like context of study, we also detected the use of *kin terms*, i.e., kinship nouns, such as nicknames, endearments, and effective references. With reference to the counting procedure of qualifying devices, we labeled each marker either as “positive” or “negative.” We coded a positive qualifying device if it was one (or more) of the following cases: (a) an augmentative adjective/locution/phrasal expression, with positive evaluation; (b) positive assessment of an adjective/locution/phrasal expression; and (c) comparative or superlative adjective with a positive evaluation. Conversely, we coded a negative qualifying device if there was: (a) a negation particle before an adjective, conveying global negative evaluation; (b) pejorative adjective/locution/phrasal expression; (c) diminutive adjective/locution/phrasal expression without positive affective tones; and (d) comparative or superlative adjective with negative evaluation. Furthermore, we coded as “residual,” each qualifying device that was not evaluated nor negative or positive. For any of the abovementioned coding and counting actions, we double-checked the operations and jointly discussed ambiguous cases.

## Results

In this section, we illustrated our results by focusing on emergent groups that professionals refer to and by displaying social categorization markers and qualifying devices used for internal and external stakeholders.

### Internal and External Stakeholders

Our analysis first shows that participants refer to numerous stakeholders, either internal or external, and with reference to past, present, and future. During the ethnographic interview, participants mentioned internal stakeholders (either groups, organizations, or couples) 668 times (refer to Supplementary Figure 1). In contrast, participants mentioned external stakeholders 254 times (refer to Supplementary Figure 2). At the first glance, we can see that professionals refer to internal stakeholders around three times more than external ones. Conversely, they mentioned a greater number of external stakeholders (15 external ones vs. 11 internal ones). When referring to external groups, professionals mentioned three different types of family, namely, the extended biological families of children in care, the adoptive and foster families where children in care can be hosted, and the families of schoolmates, and two generalized categories, namely, “external context” (e.g., “the world out there”) and “external people” (e.g., “the common sense people”), using a different granularity with respect to internal stakeholders. In fact, when talking about internal stakeholders, professionals made reference to subgroups of either past ones, present, or future ones.

### Internal Stakeholders (Ingroup)

Taking into consideration internal stakeholders, participants referred to eight groups, two couples (i.e., educator and in-house psychologist and general manager and in-house psychologist), and one organization (i.e., the congregation).

Table 1 illustrates the frequency distribution of the use of professionals of social categorization markers when talking about internal stakeholders. During the ethnographic interview, the most frequent reference has been made to children in care, to staff and children, and to the whole staff (i.e., nuns, educator, and in-house psychologist). On the contrary, the less frequent references have been made to in-house psychologists and children, auxiliary staff, and volunteers.

**TABLE 1:** Frequency and percentage of internal stakeholder (ingroup) social categorization markers.

Social categorization markers	GRE – Common nouns		GRE – Pronouns		Total for each category	Total on 668
	*f*	%	*f*	%	*f*	%
Children in care	168	*75*	55	*25*	223	*33*
Staff and children in care	126	*100*	0	*0*	126	*19*
Nuns, educator, and in-house psychologist	15	*13*	102	*87*	117	*18*
Nuns	20	*24*	62	*76*	82	*12*
Educator and psychologist	14	*23*	46	*77*	60	*9*
Future staff members	19	*100*	0	*0*	19	*3*
Congregation	11	*92*	1	*8*	12	*2*
General manager and in-house psychologist	0	*0*	10	*100*	10	*1*
Auxiliary staff	9	*100*	0	*0*	9	*1*
Volunteers	9	*100*	0	*0*	9	*1*
In-house psychologist and children	0	*0*	1	*100*	1	*0,1*
Total	391	*59*	277	*41*	668	*100*

*Italic indicates difference between frequencies and percentage values.*

In detail (Table 1), we observed that for what is concerning the use of common nouns, the most mentioned internal stakeholders were the children in care (168), the group of children and staff included (126), and nuns (20). Furthermore, kin terms were mainly used for nuns (4) (refer to Supplementary Material). For what is concerning the use of pronouns, the most mentioned internal stakeholders were the entire team (102), followed by the nuns (62) and children (55) (Table 1). Table 2 illustrates in detail the qualifying devices used by professionals when referring to internal stakeholders, i.e., ingroup.

**TABLE 2:** Frequency and percentage of internal stakeholder (ingroup) qualifying devices.

Internal stakeholders	Total		Positive		Negative		Residual	
	*f*	%	*f*	%	*f*	%	*f*	%
Children in care	47	*53*	10	*21*	21	*45*	16	*34*
Nuns	7	*8*	6	*86*	0	*0*	1	*14*
General manager and in-house psychologist	0	*0*	0	*0*	0	*0*	0	*0*
Nuns, educator and in-house psychologist	29	*33*	23	*79*	4	*14*	2	*7*
Educator and psychologist	0	*0*	0	*0*	0	*0*	0	*0*
Staff and children in care	0	*0*	0	*0*	0	*0*	0	*0*
In-house psychologist and children	0	*0*	0	*0*	0	*0*	0	*0*
Volunteers	1	*1*	1	*100*	0	*0*	0	*0*
Future staff members	1	*1*	0	*0*	0	*0*	1	*100*
Auxiliary staff	2	*2*	0	*0*	0	*0*	2	*100*
Congregation	2	*2*	0	*0*	0	*0*	2	*100*
Total *f*	89		40		25		24	
Total%	*100*		*45*		*28*		*27*	

*Italic indicates difference between frequencies and percentage values.*

Table 2 displays that internal stakeholders have been frequently qualified. The most defined ones were as follows: children (47 qualifiers); nuns, educator, and in-house psychologist (29); and nuns (7). The less defined subgroups were as follows: volunteers and future staff members (1 for both categories). There are several groups with no use of qualifying devices. In general, internal stakeholders were positively assessed in 45% of cases and negatively assessed in 28% of cases, and the residually qualified were 27%. In percentage terms, the most positively evaluated were volunteers (100%, e.g., “free”), nuns (86%, e.g., “stable”), and staff (79%, e.g., “strong”). Conversely, the most negatively qualified were the group of children (45%, e.g., “blocked,” “detached,” and “too distressed”), followed by the entire staff that has been qualified with negative terms in the 14% of cases (e.g., “not structured”).

### External Stakeholders (Outgroup)

For what is concerning external stakeholders, participants referred mainly to organizations (8) and groups (6); residually, they refer to a couple (1), i.e., a single mother and child (refer to Table 3).

**TABLE 3:** Frequency and percentage of external stakeholder (outgroup) social categorization markers.

Social categorization markers	GRE – Common nouns		GRE – Pronouns		Total for each category	Total on 254
	*f*	%	*f*	%	*f*	%
Schools	58	*100*	0	*0*	58	*23*
Children not in care at “Nun’s Home”	43	*98*	1	*2*	44	*17*
Children’s extended biological families	44	*100*	0	*0*	44	*17*
Social Services	18	*90*	2	*10*	20	*8*
Other residential care facilities	19	*100*	0	*0*	19	*7*
Health care services	18	*100*	0	*0*	18	*7*
External context	18	*100*	0	*0*	18	*7*
External people	13	*100*	0	*0*	13	*5*
Other families	8	*100*	0	*0*	8	*3*
Judicial services	4	*100*	0	*0*	4	*2*
Public Administration	2	*100*	0	*0*	2	*0,8*
Adoptive and foster families	2	*100*	0	*0*	2	*1*
University	2	*100*	0	*0*	2	*1*
Police	1	*100*	0	*0*	1	*0,4*
Mum and child dyad	0	*0*	1	*100*	1	*0,4*
Total	250	*98*	4	*2*	254	*100*

*Italic indicates difference between frequencies and percentage values.*

Table 3 illustrates the frequency distribution of the use of participants of social categorization markers when talking about external stakeholders. The most frequent references have been made to schools (58), children not in care at “Nuns’ Home” (44), extended biological families of children (44), and to social services (20). On the contrary, the less frequent references have been made to public administration (2), adoptive and foster families (2), university (2), police (1), and mother and child dyad (1). In detail (Table 3), we observed that for what is concerning GREs, almost all external groups were mentioned through common nouns (250 on 254), while kin terms (refer to Supplementary Material) and pronouns were used only residually (Table 3). Pronouns were used only for social services (2), children not in care (1), and mother and child dyad (1).

For what is concerning the frequency and use of qualifying devices, Table 4 displays that external stakeholders have been frequently qualified. The most “defined” organizations were schools (11) and healthcare services (5), while the most qualified groups were children not in care (6), extended biological families of children (7), and adoptive and foster families (3). The less defined organizations were as follows: other residential care facilities, external context, judicial services, and police (1 for both categories).

**TABLE 4:** Frequency and percentage of external stakeholder (outgroup) qualifying devices.

External stakeholders	Total		Positive		Negative		Residual	
	*f*	%	*f*	%	*f*	%	*f*	%
Schools	11	*31*	0	*0*	10	*91*	1	*9*
Children not in care at “Nun’s Home”	6	*17*	3	*50*	0	*0*	3	*50*
Children’s extended biological families	7	*19*	0	*0*	7	*100*	0	*0*
Social Services	0	*0*	0	*0*	0	*0*	0	*0*
Other residential care facilities	1	*3*	0	*0*	1	*100*	0	*0*
Health care services	5	*14*	1	*20*	0	*0*	4	*80*
External context	1	*3*	0	*0*	0	*0*	1	*100*
External people	0	*0*	0	*0*	0	*0*	0	*0*
Other families	0	*0*	0	*0*	0	*0*	0	*0*
Judicial services	1	*3*	0	*0*	1	*100*	0	*0*
Public Administration	0	*0*	0	*0*	0	*0*	0	*0*
Adoptive and foster families	3	*8*	2	*67*	1	*33*	0	*0*
University	0	*0*	0	*0*	0	*0*	0	*0*
Police	1	*3*	0	*0*	*1*	*100*	0	*0*
Mum and child dyad	0	*0*	0	*0*	0	*0*	0	*0*
Total *f*	36		6		21		9	
Total%	100		17		58		25	

*Italic indicates difference between frequencies and percentage values.*

In general, external stakeholders were negatively assessed in 58% of cases, positively in 17% of cases (while residually qualified in 25% of cases). In percentage terms, the most negatively evaluated were extended biological families of children (e.g., “very disintegrated” and “destructive”), other residential care facilities (“not protective”), judicial services, police (100% for both categories), and schools (91%, e.g., “invasive”). Conversely, the most positively qualified were the adoptive and foster families (67%, e.g., “long-lasting”), followed by healthcare services that have been qualified with positive terms in the 20% of cases (e.g., “well prepared”).

## Discussion

Differently from a traditional laboratory study on intergroup relations, our investigation has been based on a real-life community of practice of professionals that rely on – and at the same time delivers – intensive group care work. Our results illustrate that intergroup relations are locally tied and categorically permeable. First, by accounting for the multitude of discourses of ingroups and outgroups that professionals refer to, our results illustrate the complexity of different internal and external groups (mentioned as past groups, present ones, and hypothetical ones in the future). Rather than being established *a priori*, these categories appear to be permeable: interestingly, in fact, each professional include herself and other members (children included) in different ingroups at the same time, involving subgroups, couples, and organizational roles. To do so, participants explicitly rely on collective nouns, kin terms, and positive qualifications. In contrast, external stakeholders were less frequently enumerated and more negatively qualified. Very few external groups were “talked into being” by using kinship terms, and only a residual number of them were invoked with pronouns, marking their relevant social identities as distant and uniformed, as in any “othering” process, i.e., when building homogenous external groups (Lalander and Herz, 2018).

By the analysis of group qualifications, our discursive study substantially confirms the ingroup bias, promoting an internal valorization of the ingroup over the outgroup. This is particularly evident when professionals compare their work with the other residential care facilities, and when they qualify children in care and their biological families, echoing the use of “contrastive rhetoric,” frequently used in the social work field “when establish[ing] a deviant case for discredited character” (Hall et al., 2006, p. 56). Conversely, “external” substitute families, such as adoptive and foster families, appear to be more positively judged.

However, the discursive accomplishment of the ingroup bias appears to be not for free in terms of interactive work: participants use around three times more social categorization markers to refer to internal stakeholders. If on the one hand, this can be related to the institutional format of the interview, explicitly asking to make sense out of their work, on the other hand, it appears as though they need a greater amount of interactive work to justify, compare, and make distinctions over itself, opening up for internal fragmentation.

Unquestionably, however, an internal group appears to be constructed as more “familial” (see the use of kinship terms) and more positively qualified over the rest: the group of resident nuns. According to SIT, this can be explained by referring to internal prototyping (Hogg and Terry, 2000), implying a clear theory of governance, organizational and moral leadership of nuns over the rest of the team.

Additionally, by frequently relying on comparison between internal and external groups, between “good and evil,” this discursive accomplishment echoes the activity of stereotyping, usually implied when there is neither direct nor frequent interaction with the (negatively qualified) external groups. In this case, this hypothesis is not coherent with our –ethnographic observations (Saglietti, 2010, 2012, 2019) and with perspectives of participants, both documenting that “Nuns’ Home” maintains frequent and detailed interaction with most of the above-mentioned external groups. How this can be explained? Drawing on our previous research, this can be matched with their specific interactive pattern, which is centered on global interactive control and self-reference of nuns, with less possibility for anyone else to contribute (other than with resistance or unnegotiated compliance) (Saglietti and Zucchermaglio, 2021), opening for interesting research on the isomorphism on talk and (interactive) work.

## Conclusion

As many scholars “have called for research that provides a link between micro and macro issues of language use” (Maheux-Pelletier and Golato, 2008, p. 689), in this study, we attempted to illustrate many implications that a discursive investigation of the local organization of a microcosm of talk (i.e., the use of social categorization markers and qualifying devices of groups during an ethnographical interview) has to illuminate a larger social issue, such as intergroup relations. We did so by originally focusing on an intensive group-care context, i.e., group homes for out-of-home children, which has been rarely investigated by group research. Particularly, by the in-depth analysis applied to an-already-revealed-as-particularly-closed community of practices (Saglietti, 2012; Saglietti and Zucchermaglio, 2021), we illustrated that the discursive devices, “no matter how minute and apparently ‘linguistic’ in character, must be investigated as forms of social action and not simply manifestations of underlying grammatical machinery” (Goodwin and Goodwin, 1990, p. 4). In this light, this study can be considered relevant as it confirms that the discursive investigations of both social categorization markers and qualifying devices can reveal ingroup bias, intergroup relations, group qualification, and can render the multitude of groups that come into play in the work of a single organization, revealing its closeness/openness over the rest of its social world. This qualitative approach can be applied to any group-care interventions, such as schools and clinical interventions, not only for doing group research but also for training professionals working in these fields.

Limitations of this study, however, address the sampling, the limited number of the investigated discursive devices, and the analyzed discursive genre. First, drawing on a single ethnographical interview from a single community of practice, our results cannot be generalized. This study must therefore be read with keeping in mind the multiple variables depending on the interview format and on the personal features of the interviewer (i.e., genre, age, and role, to cite a few) which necessarily impact the interaction at hand. As our results come from a peculiar field, i.e., the alternative care for out-of-home children, they need to be compared with different communities of practices and other organizational sectors. Addressing these issues and by better embracing the complex discursive accomplishment of intergroup relations, our future research will then include a comparison between the different group homes belonging to our dataset (Saglietti, 2012) and an in-depth investigation of other discursive features (such as alignment and affiliation, repair, turn orchestration, speaker selection, and quotations) and participatory framework devices (such as the discursive roles of each participant) (Saglietti and Marino, in preparation^3^).

## Data Availability Statement

The original contributions presented in the study are included in the article/Supplementary Material, further inquiries can be directed to the corresponding author/s.

## Ethics Statement

The studies involving human participants were reviewed and approved by University Ethics Committee; Local Social Services of the Municipality of Rome (IT); deputy public prosecutor of the local Juvenile Court. Written informed consent to participate in this study was provided by professionals themselves. For children’s involvement, written informed consent was provided by their legal guardians. Once provided, children themselves granted their verbal assent to their research involvement.

## Author Contributions

Both authors contributed to the conception and design of the study. They worked on the analysis, wrote the manuscript, contributed to the manuscript revision, read and approved the submitted version.

## Conflict of Interest

The authors declare that the research was conducted in the absence of any commercial or financial relationships that could be construed as a potential conflict of interest.

## Publisher’s Note

All claims expressed in this article are solely those of the authors and do not necessarily represent those of their affiliated organizations, or those of the publisher, the editors and the reviewers. Any product that may be evaluated in this article, or claim that may be made by its manufacturer, is not guaranteed or endorsed by the publisher.

## Footnotes

1 Nevertheless, following the study by Schegloff (1993), we are well aware “that one can avoid using a person reference ‘filler’ and yet still achieve reference” (Wilson and Zeitlyn, 1995, p. 66).

2 In Italian, they are as follows: noi (we/us), voi (you), essi, loro (they), ci, ce (us), si, se (them), ve, vi (you), and li (them). We excluded from the dataset when these devices were part of reflexive verbs and locutions.

3 Saglietti, M., and Marino, F. (in preparation). *Intergroup Relations, Participation, Alignment and Affiliation in Group Care. An In-depht Discursive Study.*
