# Supplementary material for: Accomplishing Intergroup Relations in Group Homes: A Discursive Analysis of Professionals Talking About External and Internal Stakeholders
Source: Front Psychol. 2022 Mar 22;13:784345. doi: 10.3389/fpsyg.2022.784345 (PMC8982676; doi:10.3389/fpsyg.2022.784345)
Supplement: Supplementary file 1 [file Data_Sheet_1.docx]

**Supplemental Materials**

**Accomplishing intergroup relations in group homes: A discursive analysis of professionals talking about external and internal stakeholders**

by Marzia Saglietti and Filomena Marino

**FIG 1:** Internal stakeholders (ingroup) social categorization markers frequency.

**FIG 2:** External stakeholders (outgroup) social categorization markers frequency.

| TAB 5\| Internal stakeholders (ingroup) Kin terms frequency and percentage. Source: “Nun’s Home” data analysis. | | |
| --- | --- | --- |
| Internal stakeholders | ***f*** | **%** |
| Children in care | 0 | 0 |
| Staff and children in care | 2 | 33 |
| Nuns, educator, and in-house psychologist | 0 | 0 |
| Nuns | 4 | 67 |
| Educator and psychologist | 0 | 0 |
| Future staff members | 0 | 0 |
| Congregation | 0 | 0 |
| General manager and in-house psychologist | 0 | 0 |
| Auxiliary staff | 0 | 0 |
| Volunteers | 0 | 0 |
| In-house psychologist and children | 0 | 0 |
| Total *f* | 6 | |
| Total % | 1,5 | |

| TAB 6\|: External stakeholders (outgroup) kin terms frequency and percentage. Source: “Nun’s Home” data analysis. | | |
| --- | --- | --- |
| External stakeholders | ***f*** | **%** |
| Schools | 0 | 0 |
| Children not in care at “Nun’s Home” | 2 | 50 |
| Children’s extended biological families | 0 | 0 |
| Social Services | 0 | 0 |
| Other residential care facilities | 0 | 0 |
| Health care services | 0 | 0 |
| External context | 0 | 0 |
| External people | 0 | 0 |
| Other families | 2 | 50 |
| Judicial services | 0 | 0 |
| Public Administration | 0 | 0 |
| Adoptive and foster families | 0 | 0 |
| University | 0 | 0 |
| Police | 0 | 0 |
| Mum and child dyad | 0 | 0 |
| Total *f* | 4 | |
| Total % | 1,6 | |
